# High *BMI1* mRNA expression in peripheral whole blood is associated with favorable prognosis in advanced non-small cell lung cancer patients

**DOI:** 10.18632/oncotarget.15914

**Published:** 2017-03-06

**Authors:** Ana Koren, Matija Rijavec, Eva Sodja, Izidor Kern, Aleksander Sadikov, Viljem Kovac, Peter Korosec, Tanja Cufer

**Affiliations:** ^1^ University Clinic Golnik, Golnik, Slovenia; ^2^ University of Ljubljana, Faculty of Computer and Information Science, Ljubljana, Slovenia; ^3^ Institute of Oncology Ljubljana, Ljubljana, Slovenia

**Keywords:** non-small cell lung cancer, peripheral whole blood, *BMI1*, mRNA expression, prognosis

## Abstract

Polycomb group member protein BMI1 is involved in maintaining cell identity, proliferation, differentiation and human oncogenesis. In the present study, we determined *BMI1* mRNA expression in whole blood and evaluated the impact of the expression level on the treatment response and survival of 96 advanced NSCLC patients treated with first-line platinum-based chemotherapy. We also determined *BMI1* mRNA expression in primary tumors from 22 operable NSCLC patients treated with radical surgery. We found that compared with control subjects, *BMI1* mRNA expression in whole blood of advanced NSCLC patients was decreased (*P*<0.001). Similarly, we observed decreased *BMI1* mRNA expression in primary tumors compared to normal lungs from operable NSCLC patients (*P*=0.001). We found high *BMI1* mRNA expression in blood was associated with longer progression-free survival (PFS) (*P*=0.049) and overall survival (OS) (*P*=0.012) in advanced NSCLC patients treated with first-line platinum-based chemotherapy. However, no association between the *BMI1* mRNA level and response to chemotherapy was found (*P*=0.21). Multivariate Cox proportional hazards regression analysis showed elevated *BMI1* mRNA level in whole blood was an independent prognostic factor for longer PFS (*P*=0.012) and OS (*P*<0.001). In conclusion, *BMI1* mRNA expression in whole blood might represent a new biomarker for the diagnosis and prognosis of NSCLC.

## INTRODUCTION

Lung cancer is the most frequently diagnosed cancer and the main cause of cancer-related mortality worldwide. The disease accounts for more than 1.8 million newly diagnosed cancer cases globally (13 % of the total) and is estimated to be responsible for nearly 1.6 million deaths (19 % of the total) [1, 2]. Lung cancer prognosis remains poor, with an overall 5-year survival in Europe of only 13 % [3]. Non-small cell lung cancer (NSCLC) accounts for approximately 85 % of all lung cancer cases [4]. Regardless of the histological subtype, NSCLC represents one of the most genomically diverse cancers, which has led to the recognition of multiple clinically important biological subtypes [5]. Due to the aggressive nature of the disease and the lack of effective screening methods, most NSCLC patients are diagnosed with advanced, incurable disease, and the prognosis of patients with advanced NSCLC without recognizable oncogene drivers remains poor, with a median overall survival of only 10-14 months for patients treated with standard platinum-based chemotherapy [6].

Dissemination of cancer cells via the blood circulation is the key step in the progression of solid tumors, including NSCLC. Various studies have shown that circulating cell-free tumor nucleic acids, such as circulating tumor DNA (ctDNA), circulating RNA or microRNAs, reflect the same genetic characteristics as the primary tumor and/or metastases and may serve as non-invasive biomarkers for monitoring tumor spread and resistance to systemic treatment during the disease course [7, 8]. In addition, gene expression profiling in peripheral whole blood is frequently used to identify new biomarkers for different forms of human diseases and RNA-stabilized whole blood-based technologies have also been applied for lung cancer early detection, diagnosis and prognosis [9–12]. The main advantage of this approach is related to the presumption that RNA from dead cells is rapidly degraded by RNases; thus, most detectable transcripts are considered to originate from viable cells [13, 14]. Real-time quantitative PCR (RT-qPCR) is a powerful tool for mRNA quantification. Although RT-qPCR provides less genetic information than array [15] or sequencing [16] technologies, it is also less costly; therefore, analysis of only one to a few genes simultaneously might be applied for continuous monitoring of disease progression in a routine clinical setting [17].

B-lymphoma Moloney murine leukemia virus insertion region-1 (BMI1) is a member of the human polycomb group (PcG) proteins that maintain gene repression through chromatin modification, resulting in epigenetically silenced genes. These proteins have an essential role in maintaining cell identity, growth and differentiation [18], as reflected by the fact that an abnormal BMI1 expression pattern is linked to oncogenesis. Indeed, altered BMI1 expression has been frequently described in hematological malignancies [19–21] as well as in human solid tumors [22–25].

In our previous study, we showed that *BMI1* mRNA expression in primary NSCLC tumors is positively associated with *BMI1* mRNA expression in peripheral whole blood of operable NSCLC patients, suggesting the potential of measuring *BMI1* mRNA in whole blood as a surrogate marker of tumor progression [26]. Accordingly, the aim of our current study was to assess *BMI1* mRNA expression in whole blood and to evaluate its impact of expression levels on treatment response and prognosis in advanced NSCLC patients treated with platinum-based first-line chemotherapy. We also compared *BMI1* mRNA expression between primary tumors and normal lung tissue of operable NSCLC patients treated with radical surgery.

## RESULTS

### Patient and treatment characteristics

The clinical and treatment characteristics of 96 advanced and 22 operable NSCLC patients included in the study are summarized in Table 1. The median age of the advanced NSCLC patients was 62 years (range 39-79 years), and 57 of the 96 patients (59.4 %) were male. The majority of patients exhibited good PS (PS<2; 85/96; 88.6 %) and were current or former smokers (82/96; 85.4 %). Of the 96 patients, 65 (67.7 %) had adenocarcinoma, and 25 (26.0 %) had squamous cell carcinoma histology. All of the patients received first-line platinum-based chemotherapy. Most of the patients received the platinum-pemetrexed regimen (55/96; 57.3 %) or platinum-gemcitabine (38/96; 39.6 %), whereas others received either the platinum-taxanes (2/96; 2.1 %) or platinum-etoposide (1/96; 1.0%) chemotherapy regimen. The median number of chemotherapy cycles was 4 (range: 1-6). A complete or partial response was obtained in 42 of the 96 (43.7 %) patients. One-third of the patients (32/96; 33.3 %) received second-line systemic therapy, specifically pemetrexed, erlotinib or taxanes. The median follow-up time was 9.9 months (range: 1-31.5 months).

**Table 1:** Characteristics of 96 advanced and 22 operable non-small cell lung cancer (NSCLC) patients

Characteristics	*Advanced NSCLCN* (%)	*Operable NSCLCN* (%)
N° of patients	96	22
**Age in years: median (range)**	62 (39-79)	59 (48-77)
**Gender**		
Male	57 (59.4)	13 (59.1)
Female	39 (40.6)	9 (40.9)
**Histology**		
Adenocarcinoma	65 (67.7)	9 (40.9)
Squamous cell carcinoma	25 (26.0)	10 (45.5)
NOS	6 (6.3)	3 (13.6)
**Performance status^a^**		
0	14 (14.6)	8 (36.4)
1	71 (73.9)	14 (63.6)
≥2	11 (11.5)	
**Smoking history**		
Yes	82 (85.4)	22 (100.0)
No	14 (14.6)	
**Clinical stage**		
I		13 (59.1)
II		5 (22.7)
III		4 (18.2)
IV	96 (100.0)	
**Number of metastatic sites**		
<3	79 (82.3)	NA
≥3	17 (17.7)	
**Number of chemotherapy cycles**: median (range)	4 (1-6)	NA
**Type of chemotherapy**		NA
Platinum-Pemetrexed	55 (57.3)	
Platinum-Gemcitabine	38 (39.6)	
Platinum-Taxanes	2 (2.1)	
Platinum-Etoposide	1 (1.0)	
**Response to first-line platinum-based chemotherapy^b^**		
CR+PR	42 (43.7)	NA
SD	22 (22.9)	
PD	29 (30.2)	
**Second-line systemic therapy**		
Yes	32 (33.3)	NA
No	64 (66.7)	

*N*: number of patients; NOS: not otherwise specified; CR: complete response; PR: partial response; SD: stable disease; PD: progressive disease; NA: not applicable

^a^East Cooperative Oncology Group performance status.

^b^Objective response to treatment could be measured in 93/96 (96.8 %) of the patients.

The median age of operable NSCLC patients was 59 years (range 48-77 years), and 13 of 22 (59.1 %) were male. Nine of these 22 (40.9 %) patients had adenocarcinoma, and 10 (45.5 %) had squamous cell carcinoma histology. All patients were diagnosed with operable disease (stage I-III), had good performance status (PS≤1) and were treated with radical surgery.

### *BMI1* mRNA levels in peripheral whole blood of advanced NSCLC patients

**Figure 1:**
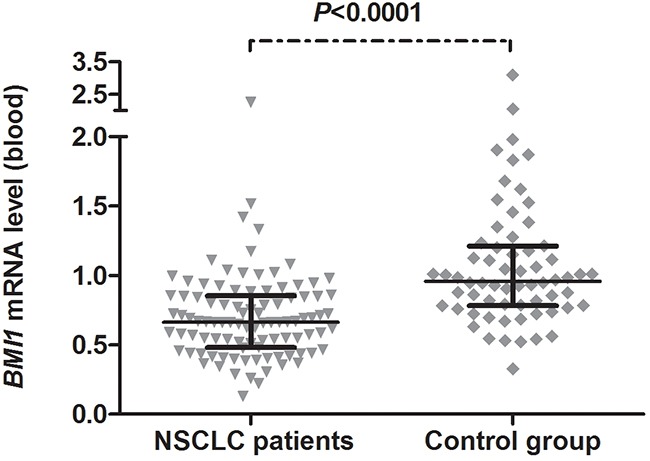
*BMI1* mRNA expression levels in peripheral blood samples of 96 advanced non-small cell lung cancer (NSCLC) patients and 64 controls Horizontal lines represent the median with interquartile range.

*BMI1* mRNA expression in whole blood of advanced NSCLC patients was significantly lower compared with the control group consisting of healthy individuals and hospital-based controls (*P*<0.0001). The median *BMI1* mRNA expression level in NSCLC patients was 0.633 (interquartile range 0.478-0.854) and in control group was 0.958 (interquartile range 0.783-1.211) (Figure 1).

### Association between blood *BMI1* mRNA levels and patient characteristics

No associations between *BMI1* mRNA expression levels and patient clinical variables or tumor characteristics, i.e., age, sex, PS, smoking status, pathological type and number of metastatic sites, were found (all *P*≥0.05, Table 2).

**Table 2:** Correlations between *BMI1* mRNA expression level and clinical characteristics of 96 advanced non-small cell lung cancer (NSCLC) patients

	***BMI1*-high *N***	***BMI1-*low *N***	***P*-value**
**Age**			
≥60	28	28	*P*=1.000^+^
<60	20	20	
**Gender**			
Female	18	21	*P*= 0.678^+^
Male	30	27	
**PS^a^**			
< 2	45	40	*P*=0.199^+^
≥ 2	3	8	
**Histology^a^**			
Adenocarcinoma	35	30	*P*=0.483^+^
Squamous cell carcinoma	11	14	
**Number of metastatic sites**			
< 3	37	42	*P*=0.285^+^
≥ 3	11	6	
**Smoking history**			
Yes	40	42	*P*=0.774^+^
No	8	6	

*N*: number of patients; ^+^Fisher's exact test.

^a^Six patients with NOS histology were excluded from the analysis.

### Association between blood *BMI1* mRNA levels and clinical outcomes

**Figure 2:**
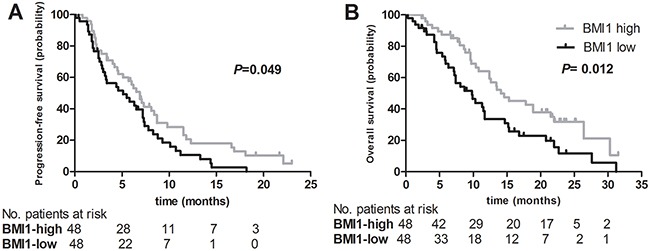
Kaplan-Meier curves for (A) progression-free survival and (B) overall survival after first-line platinum-based chemotherapy according to *BMI1* mRNA expression level in peripheral whole blood of patients with advanced non-small cell lung cancer (NSCLC)

No association between *BMI1* mRNA levels and response to first-line platinum-based chemotherapy was found (*P*=0.210; Fisher's exact test). Conversely, patients with low *BMI1* mRNA expression had a shorter median progression-free survival (PFS) (5.1 *versus* 6.9 months, *P*=0.049) and a shorter median overall survival (OS) (9.8 *versus* 14.1 months, *P*=0.012) compared with patients with high *BMI1* mRNA expression (Figure 2). Multivariate Cox proportional hazards regression analysis adjusting for age, PS and number of metastatic sites revealed that a lower *BMI1* mRNA level in whole blood is an independent prognostic factor for shorter PFS (HR=2.959; 95 % CI:1.274-6.849; *P*=0.012) and OS (HR=7.937; 95 % CI: 2.604-24.390; *P*<0.001) (Table 3).

**Table 3:** Univariate and multivariate Cox proportional hazards regression model analyses of survival

*Parameter*	Progression-free survival		Overall survival	
	UV*P*-valueHR (95% CI)	MV*P*-valueHR (95% CI)	UV*P*-valueHR (95% CI)	MV*P*-valueHR (95% CI)
***BMI1* mRNA level**	***P*=0.006**3.48 (1.44-8.40)	***P*=0.012**2.96 (1.27-6.85)	***P*<0.001**9.43 (3.13-28.57)	***P*<0.001**7.94 (2.60-24.39)
**Age** (>60 vs. ≤60)	*P*=0.1201.43 (0.91-2.25)	*P*=0.2681.29 (0.82-2.04)	*P*=0.5011.18 (0.73-1.91)	
**Gender** (M vs. F)	*P*=0.6671.09 (0.70-1.71)		*P*=0.4280.82 (0.51-1.33)	
**Histology** (AC vs. SCC)	*P*=0.5911.15 (0.69-1.91)		*P*=0.5961.16 (0.68-1.99)	
**PS^a^** (≥2 vs. <2)	***P*=0.001**3.33 (1.68-6.62)	***P*=0.007**2.62 (1.31-5.27)	***P*=0.001**3.04 (1.57-5.90)	***P*=0.021**2.22 (1.13-4.38)
***N* of metastatic sites** (≥3 vs. <3)	***P*=0.003**2.38 (1.35-4.18)	***P*=0.007**2.20 (1.24-3.91)	*P*=0.1221.64 (0.88-3.10)	***P*=0.038**1.97 (1.04-3.75)
**Smoking history** (Yes vs. No)	*P*=0.2961.43 (0.73-2.79)		*P*=0.5311.26 (0.62-2.57)	

*N*: number of patients; HR: hazard ratio; CI: confidence interval; UV: univariate analysis; MV: multivariate analysis; AC: adenocarcinoma; SCC: squamous cell carcinoma

^a^East Cooperative Oncology Group performance status

In addition, we evaluated if *BMI1* expression level has a significant association with the response to chemotherapy and survival in patients treated with different platinum chemotherapy regimens, i.e. platinum-pemetrexed (*N*=55) and platinum-gemcitabine (*N*=38). No association between *BMI1* mRNA levels and response to first-line platinum-pemetrexed (*P*=0.273) or to first-line platinum-gemcitabine (*P*=1.00) was found. Univariate Cox regression analysis of survival showed that lower *BMI1* mRNA expression is associated with shorter OS in both platinum-pemetrexed (HR:9.43; 95 % CI: 1.91-47.61; *P*=0.006) and platinum-gemcitabine (HR: 11.24; 95 % CI: 2.15-58.82; *P*=0.004) patient subgroup. It was also found that lower *BMI1* mRNA expression is associated with shorter PFS in platinum-pemetrexed (HR: 6.67; 95 % CI: 1.76-25.0; *P*=0.005) but not in platinum-gemcitabine (HR: 1.79; 95 % CI: 0.49-6.49; *P*=0.375) patient subgroup. Due to small size of subgroups reliable multivariate analysis could not be performed.

### *BMI1* mRNA levels in primary tumors of operable NSCLC patients

**Figure 3:**
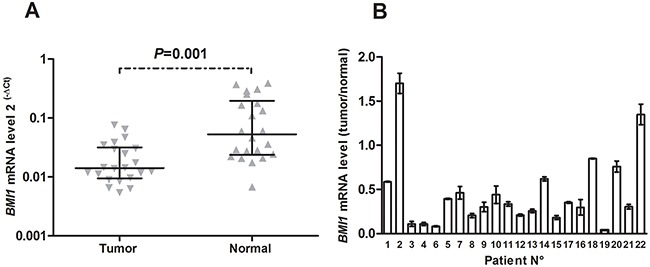
*BMI1* mRNA expression levels in 22 pairs of primary non-small cell lung cancer (NSCLC) and matching normal lung tissue samples **(A)** Horizontal lines represent the median with interquartile range. **(B)** *BMI1* mRNA expression levels from the same patient were combined together, and each bar represents the *BMI1* mRNA level in the tumor versus the adjacent normal lung tissue. The bars represent the means of 3 independent experiments ± SD.

To compare *BMI1* mRNA expression between primary tumors and normal lung tissue, *BMI1* mRNA expression levels were also measured in 22 pairs of primary NSCLC tumor and adjacent morphologically normal lung samples. We found that *BMI1* mRNA expression in primary tumors was significantly decreased compared to normal lung tissue (*P=*0.001). Overall, 90.1 % (20/22) tumors displayed decreased *BMI1* mRNA expression compared to adjacent normal lung tissue (Figure 3).

## DISCUSSION

BMI1 belongs to the PcG family of proteins, which maintain gene repression through epigenetic chromatin modifications. These proteins have an essential role in maintaining cell identity, growth and differentiation [18], which is reflected by the fact that an abnormal BMI1 expression pattern is linked to oncogenesis. To the best of our knowledge, this report is the first to describe the quantitative assessment of *BMI1* mRNA levels in whole blood from NSCLC patients and evaluating the impact of the expression level on treatment response and prognosis.

Our results showed that *BMI1* expression in whole blood of advanced NSCLC patients was decreased compared with the control group of patients. Consistent with this finding, we also found that *BMI1* expression in primary tumors of operable NSCLC patients was decreased compared with expression in adjacent normal lung tissue. This result is in agreement with the results of survival analyses, in which we confirmed the positive association between high *BMI1* expression in blood and longer PFS and OS in advanced NSCLC patients treated with platinum-based chemotherapy. The lack of association between *BMI1* expression levels and response to first-line platinum-based chemotherapy observed in our study suggests more a prognostic than a predictive value for whole blood *BMI1* expression in advanced NSCLC.

To date, results of research on BMI1 expression in lung cancer tissues are scarce and with conflicting findings. Two studies reported that BMI1 protein expression in primary tumor tissue is not a significant prognostic factor in NSCLC patients [24, 27], whereas the results of two groups showed that high BMI1 protein expression is associated with unfavorable survival of patients with operable NSCLC [28, 29]. These controversial data indicate that further exploration of the role of BMI1 in lung cancer progression is necessary. However, it is known that decreased mRNA expression of PcG genes is associated with poorer tumor differentiation and unfavorable prognosis for NSCLC patients [30]; this result complies with our finding that decreased *BMI1* mRNA expression in peripheral whole blood of advanced patients is associated with a poor prognosis, potentially indicating the specific biological role of *BMI1* in NSCLC progression.

In patients with uterine cervical or breast cancers it has been shown that high *BMI1* mRNA expression in plasma is associated with poor survival [31, 32]. In addition, high BMI1 protein expression in primary tumors is associated with decreased survival of patients with lymphoma and in patients with liver or gastric cancers [22, 33, 34]. By contrast, the results of two large studies of breast cancer patients demonstrated that high BMI1 protein expression in the primary tumor is associated with a favorable prognosis [35, 36], and similar results were obtained in glioblastoma patients [37]. Clearly, it appears that BMI1 is differently dysregulated in different types of cancer.

The results of our study did not reveal any association between *BMI1* expression levels in whole blood and several clinico-pathological characteristics of patients (i.e., age, sex, PS, smoking status, pathological type). Unexpectedly, we also did not find any correlation between *BMI1* expression levels and the number of metastatic sites, which could be explained by the low number of patients contained in the subgroup with more than three metastatic sites. This observation is similar to the result of Silva et al., who also failed to identify a correlation between *BMI1* mRNA expression levels in the plasma of 111 patients with breast cancer and the disease stage and presence of metastases [32]. By contrast, Zhang et al. did confirm a positive association between a high plasma *BMI1* mRNA level and the stage and spread of the disease in patients with uterine cervical cancer [31]. We also found no significant differences in *BMI1* expression levels among patients with adenocarcinoma and squamous cell carcinoma histology. We could find no study in the literature comparing whole blood *BMI1* expression levels between individual histological subtypes of lung cancer. However, the results of one study performed on tumor tissue samples indicated no significant difference in BMI1 protein expression between adenocarcinoma and squamous cell carcinoma [29].

Platinum-based chemotherapies have long been used as a standard treatment in NSCLC. However, resistance to treatment is a major problem that restricts the efficacy of platinum-doublets. Recent *in vitro* studies had pointed out the possible involvement of BMI1 in the platinum and gemcitabine chemoresistance: Su et al. showed that reduced BMI1 expression induced cisplatin resistance by negatively regulating BMI1-ABCG2 signaling [38]. It has also been shown that BMI1 regulates intra-tumor RRM1 levels, which are predictive of gemcitabine therapeutic efficacy [39]. We therefore expected that *BMI1* blood levels could be predictive for chemotherapy response. However, we did not find any association between *BMI1* and response to first-line platinum-based chemotherapy. This association was also not found in the patient subgroup that received platinum-gemcitabine or the subgroup that received platinum-pemetrexed. Conversely, we confirmed the association between higher *BMI1* level and better overall survival in both platinum-gemcitabine and platinum-pemetrexed subgroups of patients. Therefore, the association between BMI1 and survival does not seem to be predictive but prognostic and is not dependent on type of platinum-based doublet regimen.

In the present study, we used RT-qPCR for quantitative determination of *BMI1* mRNA expression in blood and primary tumor samples. To the best of our knowledge, this study is the first to assess *BMI1* mRNA expression levels in peripheral whole blood of patients with lung cancer. In the absence of any meaningful or predefined cut-off, we selected the observed median value as the cut-off; thus having a benefit in well balanced subgroups for analyses. Where possible (e.g. Cox regression) though, we used a continuous value of *BMI1* mRNA expression level. However, in future studies, it will be necessary to identify and validate a better threshold for distinguishing between high and low *BMI1* expression. Moreover, the majority of studies performed using primary tumors samples have applied IHC for determining BMI1 expression. Compared to the semi-quantitative protein expression scoring by IHC, the main advantage of RT-qPCR is its ability to quantitate gene expression analysis. It has been speculated that because the BMI1 IHC signal is high in most cells, this could affect detection rates, precluding the reliable categorization of samples based on IHC [36].

The detection of different splice variants could lead to contradicting data on *BMI1* expression. It is known there are four transcript/splice variants of BMI1 gene, leading to different protein products (spanning from 12.2-36.9 kDa). The 36.9 kDa transcript is the only one to have the RING and the HT domain, which were shown to be necessary for the oncogenic activity of the BMI1 [40, 41]. The Taqman assay (Hs00180411_m1) used in our study was selected to primarily target 36.9 kDa protein (BMI1-001). This assay can also detect 17.4 kDa (BMI1-002) and 12.2 kDa (BMI1-008) splice variants. In the study performed by Zhang et al. [31], primers for the detection of BMI1 gene also detect the same three transcripts and they demonstrated the association between increased circulating *BMI1* mRNA and decreased survival of patients. However, this study was done in uterine cervical cancer and not in NSCLC as was the case for our study. An alternative method to confirm BMI1 expression in lung cancer tissue could be IHC. According to the Human Protein Atlas (http://www.proteinatlas.org), BMI1 protein expression in lung and respiratory epithelial cells is medium-high. On the other hand, in lung cancer tissue the BMI1 protein expression seem to be more variable and dependent on type of anti-BMI1 antibodies used for detection (HPA030472 antibody is much more sensitive as CAB011120 antibody). With reference to the above and maybe also in spite of splice variants, the data on BMI1 expression (RNA and protein) in lung cancer tissue need further consideration, which should also include the use of more standardize primers or antibodies for the detection of BMI1. There might also be some biological difference between protein and mRNA expression, possibly due to posttranscriptional modifications. Large scale genomic projects, such is The Cancer Genome Atlas (TCGA) could provide additional *BMI1* expression data in lung cancer patients. However, the results of our limited search (data not shown) of *BMI1* mRNA expression using cBioPortal for Cancer Genomics (http://cbioportal.org) did not reveal any association between *BMI1* expression in lung cancer tissue and clinical parameters or survival. Nevertheless, additional in-depth analysis may provide more useful information.

BMI1 is an important regulator of cell proliferation. Identification of factors modulating BMI1 expression has generated major clinical interest. There are several mechanisms proposed which could affect BMI1 expression: A very recent report showed C/EBPα protein contributes to inhibit BMI1 expression, suggesting anti-BMI1 inhibition may provide new therapeutic option for lung adenocarcinoma patients with low C/EBPα and high BMI1 expression [42]. It is also known that polycomb gene with tumor suppressor activity MEL-18 down regulates BMI1 expression in breast and gastric carcinoma [43]. Other studies show *BMI1* copy number in NSCLC is unchanged [27], whereas chromosomal aberrations that may result in up-regulation of BMI1 were shown in leukemia [44]. It is evident that *BMI1* gene dosage is a critical checkpoint that lung cells must overcome to achieve transformation, but nevertheless, mechanisms controlling BMI1 regulation in lung cancer and other epithelial tumors remain un-identified.

In conclusion, the results of our study showed that *BMI1* mRNA expression levels in whole blood of NSCLC patients were decreased compared with controls and had an independent prognostic value for patients with advanced NSCLC treated with platinum-based chemotherapy. Therefore, the level of *BMI1* mRNA expression might be used as new non-invasive prognostic marker. Importantly, patients with high *BMI1* expression had better prognosis compared with patients with low *BMI1* expression. Consistent with this finding, we showed that *BMI1* expression in NSCLC primary tumors was reduced compared to normal lung tissue, together suggesting that reduced *BMI1* expression is associated with NSCLC oncogenesis. Additional studies are needed to gain more insight into the molecular mechanisms of BMI1 regulation, to explain the role of BMI1 in the course of the disease, further confirm its utility as a biomarker for diagnosis and prognosis and, finally, to define its potential as a possible target for novel treatments.

## MATERIALS AND METHODS

### Patients and collection of samples

Ninety-six consecutive patients with chemotherapy-naïve EGFR mutation-negative advanced NSCLC who were treated with standard first-line platinum-based chemotherapy were enrolled for determining *BMI1* mRNA expression levels in peripheral whole blood. Twenty-two consecutive patients with operable NSCLC who were treated with radical surgery were enrolled for evaluating *BMI1* mRNA expression levels in tumors and in matching morphologically normal lung tissue. Patients with a history of other malignancies were excluded. All patients enrolled in this study had pathologically confirmed NSCLC and were treated and followed-up at University Clinic Golnik or Institute of Oncology Ljubljana. Platinum-based schemas were used in standard dosages, determined in the pivotal registration trials and recommended by the drugs labeling and clinical guidelines (https://www.nccn.org). Accordingly, the majority of patients with non-squamous-cell histology received platinum-pemetrexed while all patients with squamous-cell histology received platinum-gemcitabine chemotherapy regimen. Tumor response to platinum-based chemotherapy was evaluated according to Response Evaluation Criteria In Solid Tumors (RECIST), version 1.1 [45]. The duration of treatment was according to the standard clinical guidelines, i.e. until progression (by RECIST) or unacceptable toxicity. Data were collected for age, sex, performance status [46], smoking status, sites of metastases, type of chemotherapy and number of cycles received, response to chemotherapy and survival. Additionally, 36 healthy blood donors and 28 hospital-based controls with no history of malignant disease, matched with patients according to sex and age, were selected. The hospital-based controls included individuals suffering from advanced chronic obstructive pulmonary disease (COPD) (7), asthma (10) and sarcoidosis (11).

Whole blood (2.5 ml) was collected before the first cycle of chemotherapy (patients) or at routine examination (controls) into PAXgene blood tubes (PreAnalytiX GmbH, Hombrechtikon, Switzerland), containing a proprietary solution that reduces RNA degradation and gene induction [47]. To avoid contamination of the blood sample with skin cells, all blood samples were obtained after the first 5 ml of blood was discarded; samples were stored at −40°C until RNA isolation. Tissue samples were obtained immediately after surgery by a pathologist and stored in RNAlater (Qiagen GmbH, Hilden, Germany) at − 40°C until RNA isolation.

This study was approved by the Slovenian National Committee for Medical Ethics, protocol number 40/04/12. Written inform consent was obtained from each participant before entering the study.

### RNA isolation and cDNA synthesis

Total RNA from tumor and normal tissue samples was extracted using a miRNeasy Mini Kit (Qiagen) according to the manufacturer's instructions. RNA from peripheral whole blood samples was extracted using a PAXgene Blood miRNA Kit (PreAnalytiX) as previously described [48]. Briefly, all RNA samples were treated with RNase-free DNase (Qiagen) and reverse transcribed to cDNA using high-capacity cDNA Reverse Transcription Kit (Applied Biosystems, Foster City, CA, USA).

### RT-qPCR

Quantification of cDNA was performed by RT-qPCR (ABI PRISM 7500 Real-Time PCR System; Applied Biosystems) under standard conditions utilizing TaqMan Universal PCR Master Mix II (Applied Biosystems). TaqMan assay BMI1 (Hs00180411_m1) was utilized to quantify *BMI1* mRNA expression levels. All measurements were performed in triplicate for each sample, and relative expression was analyzed using the ΔΔCt method [49]. Through this method, the amounts of target gene mRNA were normalized to an endogenous control and related to a calibrator sample using the formula RQ sample= 2^−(ΔCt sample-ΔCt calibrator)^. Glyceraldehyde-3-phosphate dehydrogenase (GAPDH; 4333764) was used as an endogenous control (Applied Biosystems). A pooled control sample generated by mixing RNA obtained from blood samples from 10 healthy controls at equal concentrations was used as a calibrator.

### Statistical analyses

Comparison of *BMI1* mRNA expression levels between groups (controls, normal lung) was assessed by the Mann-Whitney U test or Wilcoxon test, as appropriate. The relationship between *BMI1* mRNA expression levels and patient characteristics was evaluated using Fisher's exact test. OS was defined as the period of time in months from the date of diagnosis to the date of death; PFS was defined as the period of time in months from the beginning of platinum-based chemotherapy to the date of progression or death. The optimal cut-off value between low and high *BMI1* mRNA expression level was set at the median (0.663). Survival probabilities, OS and PFS, were estimated by the Kaplan-Meier method, and the log-rank test was used to compare different categories. The Cox proportional hazards regression model was employed to identify the prognostic value of continuously distributed *BMI1* mRNA expression levels in univariate and multivariate analysis. A *P*-value below 0.05 was considered to be statistically significant. All statistical analyses were carried out using SPSS (version 21, Chicago, IL, USA) and GraphPad Prism software (version 5, San Diego, CA, USA). All reported *P*-values are two-tailed.

## References

[1] Ferlay J, Soerjomataram I, Dikshit R, Eser S, Mathers C, Rebelo M, Parkin DM, Forman D, Bray F (2015). Cancer incidence and mortality worldwide: Sources, methods and major patterns in GLOBOCAN 2012. Int J Cancer.

[2] Ferlay J, Soerjomataram I, Ervik M, Dikshit R, Eser S, Mathers C, Rebelo M, Parkin D, Forman D, Bray F (2013.). GLOBOCAN 2012 v1.0, Cancer Incidence and Mortality Worldwide: IARC CancerBase No. 11 [Internet].

[3] De Angelis R, Sant M, Coleman MP, Francisci S, Baili P, Pierannunzio D, Trama A, Visser O, Brenner H, Ardanaz E, Bielska-Lasota M, Engholm G, Nennecke A (2013). Cancer survival in Europe 1999-2007 by country and age: results of EUROCARE-5-a population-based study. Lancet Oncol.

[4] Collins LG, Haines C, Perkel R, Enck RE (2007). Lung cancer: diagnosis and management. Am Fam Physician.

[5] Li T, Kung HJ, Mack PC, Gandara DR (2013). Genotyping and genomic profiling of non–small-cell lung cancer: Implications for current and future therapies. J Clin Oncol.

[6] Cufer T, Knez L (2014). Update on systemic therapy of advanced non-small-cell lung cancer. Expert Rev Anticancer Ther.

[7] Taenzer A, Alix-Panabières C, Wikman H, Pantel K (2012). Circulating tumor-derived biomarkers in lung cancer. J Thorac Dis.

[8] Taniguchi K, Uchida J, Nishino K, Kumagai T, Okuyama T, Okami J, Higashiyama M, Kodama K, Imamura F, Kato K (2011). Quantitative detection of EGFR mutations in circulating tumor DNA derived from lung adenocarcinomas. Clin Cancer Res.

[9] Zander T, Hofmann A, Staratschek-Jox A, Classen S, Debey-Pascher S, Maisel D, Ansén S, Hahn M, Beyer M, Thomas RK, Gathof B, Mauch C, Delank KS (2011). Blood-based gene expression signatures in non-small cell lung cancer. Clin Cancer Res.

[10] Moss AC, Jacobson GM, Walker LE, Blake NW, Marshall E, Coulson JM (2009). SCG3 transcript in peripheral blood is a prognostic biomarker for REST-deficient small cell lung cancer. Clin Cancer Res.

[11] Rotunno M, Hu N, Su H, Wang C, Goldstein AM, Bergen AW, Consonni D, Pesatori AC, Bertazzi PA, Wacholder S, Shih J, Caporaso NE, Taylor PR (2011). A gene expression signature from peripheral whole blood for stage I lung adenocarcinoma. Cancer Prev Res.

[12] Sodja E, Rijavec M, Koren A, Sadikov A, Korošec P, Cufer T (2016). The prognostic value of whole blood SOX2, NANOG and OCT4 mRNA expression in advanced small-cell lung cancer. Radiol Oncol.

[13] Tsui NBY, Ng EKO, Lo YMD (2002). Stability of endogenous and added RNA in blood specimens, serum, and plasma. Clin Chem.

[14] Saloustros E, Perraki M, Apostolaki S, Kallergi G, Xyrafas A, Kalbakis K, Agelaki S, Kalykaki A, Georgoulias V, Mavroudis D (2011). Cytokeratin-19 mRNA-positive circulating tumor cells during follow-up of patients with operable breast cancer: prognostic relevance for late relapse. Breast Cancer Res.

[15] Vartanian K, Slottke R, Johnstone T, Casale A, Planck SR, Choi D, Smith JR, Rosenbaum JT, Harrington CA (2009). Gene expression profiling of whole blood: comparison of target preparation methods for accurate and reproducible microarray analysis. BMC Genomics.

[16] Wang Z, Gerstein M, Snyder M (2009). RNA-Seq: a revolutionary tool for transcriptomics. Nat Rev Genet.

[17] Hantzsch M, Tolios A, Beutner F, Nagel D, Thiery J, Teupser D, Holdt LM (2014). Comparison of whole blood RNA preservation tubes and novel generation RNA extraction kits for analysis of mRNA and miRNA profiles. PLoS One.

[18] Levine SS, King IFG, Kingston RE (2004). Division of labor in Polycomb group repression. Trends Biochem Sci.

[19] van Kemenade FJ, Raaphorst FM, Blokzijl T, Fieret E, Hamer KM, Satijn DP, Otte AP, Meijer CJ (2001). Coexpression of BMI-1 and EZH2 polycomb-group proteins is associated with cycling cells and degree of malignancy in B-cell non-Hodgkin lymphoma. Blood.

[20] Beà S, Tort F, Pinyol M, Puig X, Hernández L, Hernández S, Fernandez PL, van Lohuizen M, Colomer D, Campo E (2001). BMI-1 gene amplification and overexpression in hematological malignancies occur mainly in mantle cell lymphomas. Cancer Res.

[21] Raaphorst FM, van Kemenade FJ, Blokzijl T, Fieret E, Hamer KM, Satijn DP, Otte AP, Meijer CJ (2000). Coexpression of BMI-1 and EZH2 polycomb group genes in Reed-Sternberg cells of Hodgkin’s disease. Am J Pathol.

[22] Liu JH, Song LB, Zhang X, Guo BH, Feng Y, Li XX, Liao WT, Zeng MS, Huang KH (2008). Bmi-1 expression predicts prognosis for patients with gastric carcinoma. J Surg Oncol.

[23] Mihic-Probst D, Kuster A, Kilgus S, Bode-Lesniewska B, Ingold-Heppner B, Leung C, Storz M, Seifert B, Marino S, Schraml P, Dummer R, Moch H (2007). Consistent expression of the stem cell renewal factor BMI-1 in primary and metastatic melanoma. Int J Cancer.

[24] Kikuchi J, Kinoshita I, Shimizu Y, Kikuchi E, Konishi J, Oizumi S, Kaga K, Matsuno Y, Nishimura M, Dosaka-Akita H (2010). Distinctive expression of the polycomb group proteins Bmi1 polycomb ring finger oncogene and enhancer of zeste homolog 2 in non-small cell lung cancers and their clinical and clinicopathologic significance. Cancer.

[25] Guo BH, Feng Y, Zhang R, Xu LH, Li MZ, Kung HF, Song LB, Zeng MS (2011). Bmi-1 promotes invasion and metastasis, and its elevated expression is correlated with an advanced stage of breast cancer. Mol Cancer.

[26] Koren A, Rijavec M, Kern I, Sodja E, Korosec P, Cufer T (2016). BMI1, ALDH1A1, and CD133 transcripts connect epithelial-mesenchymal transition to cancer stem cells in lung carcinoma. Stem Cells Int.

[27] Vonlanthen S, Heighway J, Altermatt HJ, Gugger M, Kappeler A, Borner MM, van Lohuizen M, Betticher DC (2001). The bmi-1 oncoprotein is differentially expressed in non-small cell lung cancer and correlates with INK4A-ARF locus expression. Br J Cancer.

[28] Zhang X, Sun J, Wang H, Lou Y, Zhang Y, Sha H, Feng J, Han B (2014). IGF-1R and Bmi-1 expressions in lung adenocarcinoma and their clinicopathologic and prognostic significance. Tumor Biol.

[29] Vrzalikova K, Skarda J, Ehrmann J, Murray PG, Fridman E, Kopolovic J, Knizetova P, Hajduch M, Klein J, Kolek V, Radova L, Kolar Z (2008). Prognostic value of Bmi-1 oncoprotein expression in NSCLC patients: a tissue microarray study. J Cancer Res Clin Oncol.

[30] Hassan KA, Chen G, Kalemkerian GP, Wicha MS, Beer DG (2009). An embryonic stem cell-like signature identifies poorly differentiated lung adenocarcinoma but not squamous cell carcinoma. Clin Cancer Res.

[31] Zhang X, Wang C, Wang L, Du L, Wang S, Zheng G, Li W, Zhuang X, Zhang X, Dong Z (2012). Detection of circulating Bmi-1 mRNA in plasma and its potential diagnostic and prognostic value for uterine cervical cancer. Int J Cancer.

[32] Silva J, García V, García JM, Peña C, Domínguez G, Díaz R, Lorenzo Y, Hurtado A, Sánchez A, Bonilla F (2007). Circulating Bmi-1 mRNA as a possible prognostic factor for advanced breast cancer patients. Breast Cancer Res.

[33] Raaphorst FM, Otte AP, van Kemenade FJ, Blokzijl T, Fieret E, Hamer KM, Satijn DPE, Meijer CJLM (2001). Distinct BMI-1 and EZH2 Expression patterns in thymocytes and mature T cells suggest a role for Polycomb genes in human T Cell differentiation. J Immunol.

[34] Wang H, Pan K, Zhang H, Weng D, Zhou J, Li J, Huang W, Song H, Chen M, Xia J (2008). Increased polycomb-group oncogene Bmi-1 expression correlates with poor prognosis in hepatocellular carcinoma. J Cancer Res Clin Oncol.

[35] Choi YJ, Choi YL, Cho EY, Shin YK, Sung KW, Hwang YK, Lee SJ, Kong G, Lee JE, Kim JS, Kim JH, Yang JH, Nam SJ (2009). Expression of Bmi-1 protein in tumor tissues is associated with favorable prognosis in breast cancer patients. Breast Cancer Res Treat.

[36] Pietersen AM, Horlings HM, Hauptmann M, Langerød A, Ajouaou A, Cornelissen-Steijger P, Wessels LF, Jonkers J, van de Vijver MJ, van Lohuizen M (2008). EZH2 and BMI1 inversely correlate with prognosis and TP53 mutation in breast cancer. Breast Cancer Res.

[37] Cenci T, Martini M, Montano N, D`Alessandris QG, Falchetti ML, Annibali D, Savino M, Bianchi F, Pierconti F, Nasi S, Pallini R, Larocca LM (2012). Prognostic relevance of c-Myc and BMI1 expression in patients with glioblastoma. Am J Clin Pathol.

[38] Su J, Wu S, Tang W, Qian H, Zhou H, Guo T (2015). Reduced SLC27A2 induces cisplatin resistance in lung cancer stem cells by negatively regulating Bmi1-ABCG2 signaling. Mol Carcinog.

[39] Zhang Y, Li X, Chen Z, Bepler G (2014). Ubiquitination and degradation of ribonucleotide reductase M1 by the polycomb group proteins RNF2 and Bmi1 and cellular response to gemcitabine. PLoS One.

[40] Alkema MJ, Jacobs H, van Lohuizen M, Berns A (1997). Pertubation of B and T cell development and predisposition to lymphomagenesis in Emu Bmi1 transgenic mice require the Bmi1 RING finger. Oncogene.

[41] Li Z, Cao R, Wang M, Myers MP, Zhang Y, Xu RM (2006). Structure of a Bmi-1-Ring1B polycomb group ubiquitin ligase complex. J Biol Chem.

[42] Yong KJ, Basseres DS, Welner RS, Zhang WC, Yang H, Yan B, Alberich-Jorda M, Zhang J, de Figueiredo-Pontes LL, Battelli C, Hetherington CJ, Ye M, Zhang H (2016). Targeted BMI1 inhibition impairs tumor growth in lung adenocarcinomas with low CEBP expression. Sci Transl Med.

[43] Guo WJ, Datta S, Band V, Dimri GP (2007). Mel-18, a polycomb group protein, regulates cell proliferation and senescence via transcriptional repression of Bmi-1 and c-Myc oncoproteins. Mol Biol Cell.

[44] Larmonie NSD, Dik WA, Beverloo HB, van Wering ER, van Dongen JJM, Langerak AW (2008). BMI1 as oncogenic candidate in a novel TCRB-associated chromosomal aberration in a patient with TCRgammadelta+ T-cell acute lymphoblastic leukemia. Leukemia.

[45] Eisenhauer EA, Therasse P, Bogaerts J, Schwartz LH, Sargent D, Ford R, Dancey J, Arbuck S, Gwyther S, Mooney M, Rubinstein L, Shankar L, Dodd L (2009). New response evaluation criteria in solid tumours: revised RECIST guideline (version 1.1). Eur J Cancer.

[46] Oken MM, Creech RH, Tormey DC, Horton J, Davis TE, McFadden ET, Carbone PP (1982). Toxicity and response criteria of the Eastern Cooperative Oncology Group. Am J Clin Oncol.

[47] Rainen L, Oelmueller U, Jurgensen S, Wyrich R, Ballas C, Schram J, Herdman C, Bankaitis-davis D, Nicholls N, Trollinger D, Tryon V (2002). Stabilization of mRNA Expression in Whole Blood Samples. Clin Chem.

[48] Koren A, Sodja E, Rijavec M, Jez M, Kovac V, Korosec P, Cufer T (2015). Prognostic value of cytokeratin-7 mRNA expression in peripheral whole blood of advanced lung adenocarcinoma patients. Cell Oncol.

[49] Livak KJ, Schmittgen TD (2001). Analysis of relative gene expression data using real-time quantitative PCR and the 2(-delta delta C(T)) method. Methods.

